# Correlation Analysis between Macular Choroidal Thickness and Visual Field Mean Defect in Primary Open-Angle Glaucoma

**DOI:** 10.1155/2021/5574950

**Published:** 2021-04-09

**Authors:** Fan Li, Yiming Huo, Lihua Ma, Guangxian Tang

**Affiliations:** ^1^Department of Ophthalmology, Shijiazhuang People's Hospital, Shijiazhuang, Hebei 050000, China; ^2^Hebei Medical University, Shijiazhuang, Hebei 050000, China

## Abstract

**Purpose:**

To evaluate the differences in macular choroidal thickness and volume between patients with primary open-angle glaucoma (POAG) and healthy controls to assess the correlation between macular choroidal thickness and visual field mean defect (MD).

**Methods:**

Case-control study. A total of 101 patients (101 eyes) with POAG who were hospitalized in our hospital and 102 healthy subjects (102 eyes) matched by age, sex, and axial length were consecutively selected as the POAG group and the control group, respectively. The macular choroidal thickness and volume in nine regions were measured in all subjects by enhanced-depth imaging optical coherence tomography (EDI-OCT).

**Results:**

The mean thicknesses of the choroid in the macular area in the POAG group and healthy group were 207.97 ± 62.83 *µ*m and 208.24 ± 47.97 *µ*m, and the mean volumes were 0.63 ± 0.19 *µ*m^3^ and 0.64 ± 0.14 *µ*m^3^. There were no significant differences in macular choroidal thickness, volumes of various macular regions, or mean choroidal thickness or volume between the POAG and healthy groups (all *p* > 0.05). The macular choroidal thickness of various macular regions was not correlated with visual field MD in the POAG group (all *p* > 0.05).

**Conclusion:**

The macular choroidal thicknesses and volumes in POAG patients were not significantly different from those in healthy individuals. The macular choroidal thickness was not correlated with MD in POAG patients. Therefore, macular choroidal thickness is not an appropriate parameter to evaluate damage caused by POAG, and the role of the macular choroid thickness in POAG needs to be further investigated.

## 1. Introduction

Glaucoma is the leading cause of irreversible vision loss worldwide [1, 2]. Quigley [3] estimated that the number of glaucoma patients would increase to 79.6 million by 2020. Glaucoma is characterized by optic atrophy, visual field defects, and eventual progression to blindness. Glaucoma can be classified as open-angle glaucoma or closed-angle glaucoma [4]. Primary open-angle glaucoma (POAG) is the most common form, accounting for approximately 74% of all glaucoma patients [5].

The factors affecting glaucoma progression are not fully understood, but intraocular pressure (IOP) is considered the most important risk factor for glaucoma. Although reducing the IOP can slow or prevent disease progression, in some cases, glaucomatous optic nerve damage can be further aggravated even if the IOP is controlled, indicating that factors other than IOP may play important roles in glaucoma progression [6–10].

A study of choroidal hemodynamics in glaucoma suggested that abnormal choroidal blood flow may be one of the pathogenic mechanisms of POAG [11]. The choroid is the vascular layer located under the retina and has the highest perfusion rate of all blood vessels in the human body. Because of its importance for ocular blood flow, the choroid plays a critical role in glaucoma development and progression [12]. The thickness of the choroid is proportional to its blood flow, and choroidal thickness measurements can provide important information on choroidal blood flow velocity.

Previous studies of the relationship between POAG and choroidal thickness have generated conflicting results. Some studies [13] have found that the choroidal thickness in the eyes is thinner in subjects with advanced POAG than in healthy subjects, while other studies [14–17] have shown no difference between the two. Furthermore, other studies [18] have suggested that the choroid is thicker in patients with POAG. Most previous studies have analyzed the choroidal thickness in the fovea of the macula and/or at a location within 3 mm on the nasal or temporal side of the fovea. We believe that obtaining choroidal thickness in this manner has some limitations. Glaucoma is generally characterized by optic disc changes accompanied by corresponding optic nerve and visual field defects. The initial changes often occur at the lower edge of the optic disc, corresponding to an upper visual field defect that cannot exceed the midline; therefore, the normal retinal nerve fiber layer is symmetrically distributed in the upper and lower visual fields. Because the inner retina and ganglion cells are pushed to the foveal clivus, there is no nerve fiber layer in the fovea. Therefore, the use of a point located in the fovea or a certain position 3 mm or less from the nasal or temporal side of the fovea as the measurement point may not be suitable for evaluating the relationship between glaucoma and choroidal thickness in the macular area. Thus, there is an urgent need to develop a method that can more comprehensively and objectively assess the choroidal thickness in the entire macular area to more accurately evaluate glaucoma progression [19]. Spectral domain optical coherence tomography (SD-OCT) with enhanced-depth imaging (EDI) allows in vivo imaging of the choroid, and it can successfully obtain the mean choroidal thickness [20]. In this study, the macula was divided into nine regions, EDI-OCT was used to measure and compare the mean choroidal thickness and volume of the nine regions of the macula in POAG patients and healthy subjects in the Chinese population, the pattern of changes in macular choroidal thickness in eyes with POAG was investigated, and the role of the choroid in POAG progression was analyzed.

## 2. Materials and Methods

A total of 101 patients (101 eyes) with POAG who were hospitalized in our hospital between October 2016 and October 2019 were consecutively selected as the POAG group, and another 102 healthy subjects (102 eyes) matched by sex, age, and axial length were selected as the healthy control group. There was no significant difference in age, sex, or axial length between the two groups (Table 1).

The inclusion criteria were a diagnosis of POAG, an IOP > 21 mmHg, and age older than 40 years. The exclusion criteria were other types of glaucoma (such as closed-angle glaucoma or secondary glaucoma); previous history of ocular surgery or ocular trauma; other ophthalmic diseases, such as corneal opacity, lens opacity, or other ocular diseases affecting the examination; retinal or macular diseases of the fundus (diabetic retinopathy, retinal vein occlusion, hypertensive retinopathy, macular edema, epimacular membrane, macular degeneration, and macular hole); a diopter of spherical equivalent > ± 6.0 *D* or cylinder > ± 3.0 *D*; and systemic diseases such as diabetes and hypertension. This study was conducted according to the principles set forth in the Declaration of Helsinki and was approved by the ethics committee of Shijiazhuang People's Hospital. All subjects or their guardians signed informed consent forms.

### 2.1. OCT Examinations

**Figure 1:**
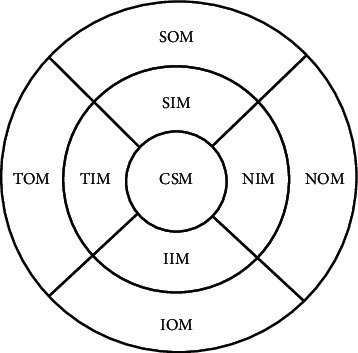
Illustration of the measurement of macular choroidal thickness at nine locations. (reproduced from Fan Li et al. [21])_._ CSM, central subfield macula; NIM, nasal inner macula; SIM, superior inner macula; IIM, inferior inner macula; TIM, temporal inner macula; NOM, nasal outer macula; SOM, superior outer macula; IOM, inferior outer macula; TOM, temporal outer macula.

**Figure 2:**
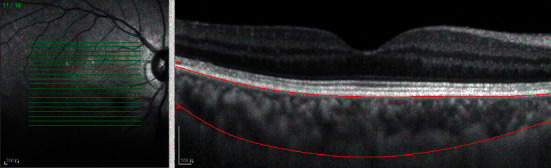
Optical coherence tomographic image (enhanced-depth imaging mode) for measurement of the macular choroidal thickness.

All subjects underwent SD-OCT (Spectralis HRA + OCT, Heidelberg, Germany). The macular thickness and volume were scanned using the EDI-OCT macular thickness map examination procedure. The specific measurement methods were as described previously [21]. The measurement of macular choroidal thickness is illustrated in Figure 1. On each scanned image, the inscribed segmentation line was labeled on the retinal pigment epithelium/Bruch membrane interface to represent the internal choroidal boundary, and the outer segmentation line was placed on the scleral/choroidal interface to represent the external choroidal boundary, as shown in Figure 2. The choroidal thickness measurements were performed by a single technician.

### 2.2. Visual Field Examinations

The visual fields of all subjects were examined using the SITA-Fast 30–2 examination procedure and a Humphrey 750i visual field analyzer (Carl Zeiss, Germany). The reliability criteria included a fixation loss rate of <20%, a false negative rate of <15%, and a false positive rate of <15%. Individuals who did not meet the criteria were excluded. The study included patients who had at least two reliable field tests.

### 2.3. Statistical Analysis

The data were analyzed using SPSS 21.0 statistical software (IBM Corporation, Armonk, NY, USA). The measurement data were normally distributed; therefore, the choroidal thickness and volume values of POAG eyes and control eyes were compared using an independent *t*-test. The correlation between macular choroidal thickness and field mean defect (MD) was analyzed by Pearson's correlation. A value of *p* < 0.05 was considered statistically significant.

## 3. Results

In the POAG group and the control group, the mean macular (MM) choroidal thicknesses were 207.97 ± 62.83 *µ*m and 208.24 ± 47.97 *µ*m, respectively, and the mean volume was 0.63 ± 0.19 *µ*m^3^ and 0.64 ± 0.14 *µ*m^3^, respectively. There were no significant differences in the mean choroidal thickness and volume of the macular area between the two groups (*t*_thickness_ = −0.025, *t*_volume_ = −0.126, all *p* > 0.05). There were no significant differences in the thickness and volume of the central subfield macula (CSM), nasal inner macular (NIM), superior inner macula (SIM), temporal inner macula (TIM), inferior inner macula (IIM), nasal outer macula (NOM), superior outer macula (SOM), temporal outer macula (TOM), and inferior outer macula (IOM) between the two groups (*t*_thickness_ = −0.230, 0.421, −0.309, 0.359, 0.746, 0.034, −0.455, −0.479, and 0.126, all *p* > 0.05; *t*_volume_ = −0.375, 0.470, −0.287, −0.340, 0.638, 0.055, −0.458, −0.485, and 0.708, all *p* > 0.05, Table 2).

The choroidal thicknesses in the CSM, NIM, SIM, TIM, IIM, NOM, SOM, TOM, and IOM areas and the mean choroidal thickness in the macular area were not correlated with visual field defects in the POAG group (*r* = 0.026, *p*=0.796; *r* = 0.026, *p*=0.796; *r* = 0.009, *p*=0.926; *r* = 0.024, *p*=0.813; *r* = 0.070, *p*=0.490; *r* = 0.041, *p*=0.687; *r* = 0.038, *p*=0.702; *r* = 0.034, *p*=0.734; *r* = 0.055, *p*=0.583; *r* = 0.036, *p*=0.720).

## 4. Discussion

The uveal tissues of the eye (choroid, iris, and ciliary body) contain abundant large and small blood vessels, and their blood flow accounts for more than 80% of ocular blood flow. Many systemic factors can affect ocular blood flow, and glaucoma patients with a history of low systolic perfusion pressure, low systolic blood pressure, and cardiovascular diseases may present glaucoma progression [19, 22]. Choroidal vessel diameter is significantly positively correlated with choroidal thickness, and choroidal expansion and macular choroidal thickening seem to be related to glaucoma severity. The density of the choriocapillaris in the macular area is reduced in patients with glaucoma, and due to the increased perfusion gradient, compensatory dilatation of the choriocapillaris in the macular area may lead to preservation of blood flow in this area [23]. There are also many factors that affect choroidal thickness. Choroidal thickness gradually decreases with age (4 *μ*m/year) and increases with increasing axis and myopia (15 *μ*m/diopter) [16, 24–26].

Despite extensive study of the choroidal thickness in POAG patients, whether choroidal thickness is associated with glaucoma etiology or progression remains unclear, and the relationship between POAG and choroidal thickness is still controversial. Nakakura et al. [19] suggested that the full thickness of the choroid in the macular area of POAG patients was not significantly different from that of healthy subjects and was not associated with glaucomatous visual field damage. A meta-analysis by Zhang et al. [15] evaluated 22 studies that measured macular choroidal thickness using EDI-OCT in POAG patients and healthy subjects and found no significant difference between the two groups. In contrast, some studies have shown that, due to the loss of the choriocapillaris and large blood vessels, the choroidal thickness is thinner in POAG patients [27, 28]. Cennamo et al. [18] found that the choroidal thickness was greater in POAG patients than in healthy subjects and concluded that the increase in the area of the choroidal lumen was responsible for the increase in choroidal thickness and that the choroid plays an important role in the vascular physiology of POAG.

In this study, the macula was divided into nine regions, and EDI-OCT was used to measure the macular choroidal thickness and volume in POAG patients and healthy subjects. No significant differences in choroidal thickness and volume in the nine regions of the macula were observed between the two groups. In both groups, the macular choroidal thickness was greater in the inner macula than in the outer macula. In the POAG group, the distribution of choroidal thickness in the inner macula followed the order SIM > TIM > IIM > NIM, and the distribution in the outer macula followed the order SOM > IOM > TOM > NOM. In the healthy subject group, the distribution of choroidal thickness in the inner macula followed the order SIM > TIM > IIM > NIM, and the distribution in the outer macula followed the order SOM > TOM > IOM > NOM. Sacconi et al. [13] found that, after normalization by age and axis, the subfoveal and temporal choroidal thicknesses in patients with advanced POAG were thinner than those in healthy subjects. This difference occurs because the chronic and long-term course of glaucoma in patients with advanced POAG causes changes in the eyes as well as damage and diffuse loss of choroidal vessels in the macular area, ultimately leading to reduced choroidal thickness. The results of this study are similar to those of previous studies [25, 29] that have reported no statistically significant difference in macular choroidal thickness between POAG patients and healthy subjects. However, previous studies measured the choroidal thickness at a certain position in the macular area rather than measuring the mean thickness of the macular area, which presents some limitations. In addition, this study found that the choroidal thickness in each region of the macula was not correlated with the visual field MD in the POAG group.

Since the choroid is a highly dynamic vascular tissue, measurement of the choroidal thickness alone cannot fully reflect the physiological changes in the hemodynamics of the choroid in glaucoma patients. Thus, this study has some limitations. First, because there are diurnal variations in choroidal thickness [30], the thicknesses measured at different time points may differ. In this study, the participants did not undergo OCT examination at a fixed time on a certain day, and therefore, some errors may be present. Second, due to the lack of automatic measurement software, manual drawing of the choroidal edges may have introduced some measurement errors. Third, although subjects with a history of hypertension were not included, the effects of systolic and diastolic blood pressure and ocular perfusion pressure on choroidal thickness were not assessed.

## 5. Conclusion

In summary, based on anatomical measurements, the choroid does not seem to be significantly altered in POAG patients compared to healthy subjects. The choroidal thickness in each region of the macula was not correlated with the visual field MD in POAG patients, and thus, choroidal thinning may not be an important component of glaucomatous optic neuropathy. Macular choroidal thickness is not an appropriate parameter to evaluate the damage caused by POAG. The pathogenesis of POAG is probably multifactorial, with important contributions from altered ocular blood flow and vascular regulation [15]. Future studies investigating choroidal vascular flow are needed to better explore the role of the choroid in the pathophysiology of open-angle glaucoma. In particular, an understanding of the clinical impact of changes in choroidal thickness on glaucoma will require multicenter studies with large samples.

## Figures and Tables

**Table 1:** Baseline characteristics of the study groups.

Groups	Eyes (n)	Sex (*n*) M/F	Age (years)	IOP (mmHg)	AL (mm)	MD (dB)
POAG	101	48/53	65.90 ± 11.02 (44–88)	27.04 ± 9.71 (15–60)	23.30 ± 0.89	−14.40 ± 9.64
Control	102	44/58	65.98 ± 10.54 (40–85)	15.24 ± 2.47 (11–21)	23.35 ± 0.88	−1.00 ± 0.39
*χ * ^2^ * /t*		0.394	−0.052	11.895	−0.388	−14.023
*P*		0.530	0.958	<0.001	0.699	<0.001

POAG, primary open-angle glaucoma; M, male; F, female; IOP, intraocular pressure; AL, axial length; MD, mean defect.

**Table 2:** Comparisons of macular choroidal thickness by EDI-OCT in the two groups.

Regions		POAG	Control	*t*	*P*
CSM	TH, *µ*m	219.83 ± 68.55	221.84 ± 55.45	−0.230	0.818
	V, *µ*m^3^	0.17 ± 0.06	0.17 ± 0.04	−0.375	0.708

NIM	TH, *µ*m	203.11 ± 70.05	199.34 ± 56.73	0.421	0.674
	V, *µ*m^3^	0.32 ± 0.11	0.31 ± 0.09	0.470	0.639

SIM	TH, *µ*m	222.04 ± 64.70	224.59 ± 52.37	−0.309	0.758
	V, *µ*m^3^	0.35 ± 0.10	0.35 ± 0.08	−0.287	0.775

TIM	TH, *µ*m	219.77 ± 64.77	222.71 ± 50.73	−0.359	0.720
	V, *µ*m^3^	0.35 ± 0.10	0.35 ± 0.08	−0.340	0.735

IIM	TH, *µ*m	218.81 ± 73.64	211.96 ± 56.15	0.746	0.457
	V, *µ*m^3^	0.34 ± 0.12	0.34 ± 0.09	0.638	0.524

NOM	TH, *µ*m	164.51 ± 63.96	164.22 ± 55.95	0.034	0.973
	V, *µ*m^3^	0.87 ± 0.34	0.87 ± 0.30	0.055	0.956

SOM	TH, *µ*m	215.80 ± 62.50	219.35 ± 47.76	−0.455	0.650
	V, *µ*m^3^	1.14 ± 0.33	1.16 ± 0.25	−0.458	0.648

TOM	TH, *µ*m	202.31 ± 56.30	205.69 ± 43.54	−0.479	0.633
	V, *µ*m^3^	1.07 ± 0.30	1.09 ± 0.23	−0.485	0.628

IOM	TH, *µ*m	205.52 ± 68.86	204.44 ± 52.85	0.126	0.900
	V, *µ*m^3^	1.09 ± 0.37	1.08 ± 0.28	0.708	0.938

MM	TH, *µ*m	207.97 ± 62.83	208.24 ± 47.97	−0.035	0.972
	V, *µ*m^3^	0.63 ± 0.19	0.64 ± 0.14	−0.126	0.900

POAG, primary open-angle glaucoma; CSM, central subﬁeld macula; NIM, nasal inner macula; SIM, superior inner macula; IIM, inferior inner macula; TIM, temporal inner macula; NOM, nasal outer macula; SOM, superior outer macula; IOM, inferior outer macula; TOM, temporal outer macula; MM, Mean macula; TH, thickness; V, volume. The data are expressed as the mean ± standard deviation.

## Data Availability

The research data used to support the findings of this study are available from the corresponding author upon request.
